# Supplementary material for: Machine Learning Accelerates Crystallization for Structure Determination
Source: Angew Chem Int Ed Engl. 2026 May 4;65(25):e1218503. doi: 10.1002/anie.1218503 (PMC13266958; doi:10.1002/anie.1218503)

checkCIF/PLATON report

Structure factors have been supplied for datablock(s) lcz086

THIS REPORT IS FOR GUIDANCE ONLY. IF USED AS PART OF A REVIEW PROCEDURE FOR PUBLICATION, IT SHOULD NOT REPLACE THE EXPERTISE OF AN EXPERIENCED CRYSTALLOGRAPHIC REFEREE.

No syntax errors found. CIF dictionary Interpreting this report

Datablock: lcz086

Bond precision: C-C = 0.0313 A Wavelength=1.54184

Cell:	a=12.5401(2)	b=21.2666(2)	c=11.2229(1)
	alpha=90	beta=90	gamma=90
Temperature:	100 K		

	Calculated	Reported
Volume	2992.98(6)	2992.98(6)
Space group	P n a 21	P n a 21
Hall group	P 2c -2n	P 2c -2n
Moiety formula	C21 H9 Ag3 F18 N6 O S	C15 H3 Ag3 F18 N6, C6 H6 O S
Sum formula	C21 H9 Ag3 F18 N6 O S	C21 H9 Ag3 F18 N6 O S
Mr	1059.01	1059.01
Dx,g cm-3	2.350	2.350
Z	4	4
Mu (mm-1)	17.611	17.610
F000	2016.0	2016.0
F000'	2027.19	
h,k,lmax	15,27,14	15,26,14
Nref	6446[3391]	4675
Tmin,Tmax	0.125,0.060	0.273,1.000
Tmin'	0.020	

Correction method= # Reported T Limits: Tmin=0.273 Tmax=1.000
AbsCorr = MULTI-SCAN

```
Data completeness= 1.38/0.73        Theta(max)= 78.630

                                                      wR2(reflections)=
R(reflections)= 0.0770( 4247)                         0.1937( 4675)

S = 1.150                     Npar= 455
```

The following ALERTS were generated. Each ALERT has the format
test-name_ALERT_alert-type_alert-level.
Click on the hyperlinks for more details of the test.

Alert level B

```
PLAT342_ALERT_3_B Low Bond Precision on  C-C Bonds ..............    0.03133 Ang.
```

Author Response: Disordered structure.

```
PLAT971_ALERT_2_B Check Calcd Resid. Dens.  0.23Ang From C1             3.42 eA-3
```

Author Response: Possible low grade disorder/absorption effects.

Alert level C

```
PLAT042_ALERT_1_C Calc. and Reported MoietyFormula Strings  Differ     Please Check
              Calc: C21 H9 Ag3 F18 N6 O S
              Rep.: C15 H3 Ag3 F18 N6, C6 H6 O S
PLAT090_ALERT_3_C Poor Data / Parameter Ratio (Zmax > 18) ........       7.24 Note
PLAT213_ALERT_2_C Atom F00L            has ADP max/min Ratio .....        3.5 prolat
PLAT234_ALERT_4_C Large Hirshfeld Difference F008     --C019     .       0.22 Ang.
PLAT241_ALERT_2_C High   'MainMol' Ueq as Compared to Neighbors of       O00U Check
PLAT906_ALERT_3_C Large K Value in the Analysis of Variance ......      4.095 Check
PLAT911_ALERT_3_C Missing FCF Refl Between Thmin & STh/L=    0.600          3 Report
                0  2  0,  14  7  0,  10  0  6,
PLAT971_ALERT_2_C Check Calcd Resid. Dens.  1.15Ang From Ag02           1.87 eA-3
```

Author Response: Possible low grade disorder/absorption effects.

```
PLAT971_ALERT_2_C Check Calcd Resid. Dens.  1.04Ang From Ag02           1.78 eA-3
```

Author Response: Possible low grade disorder/absorption effects.

```
PLAT971_ALERT_2_C Check Calcd Resid. Dens.  1.22Ang From Ag01           1.66 eA-3
```

Author Response: Possible low grade disorder/absorption effects.

```
PLAT972_ALERT_2_C Check Calcd Resid. Dens.  0.38Ang From O00U          -1.65 eA-3
PLAT977_ALERT_2_C Check Negative Difference Density on H014      .      -0.31 eA-3
PLAT977_ALERT_2_C Check Negative Difference Density on H01F      .      -0.35 eA-3
```

Alert level G

```
PLAT002_ALERT_2_G Number of Distance or Angle Restraints on AtSite         18 Note
PLAT003_ALERT_2_G Number of Uiso or U(i,j)  Restrained non-H-Atoms         14 Report
PLAT083_ALERT_2_G SHELXL Second Parameter in WGHT  Unusually Large     100.34 Why ?
PLAT142_ALERT_4_G s.u. on b - Axis Small or Missing .............    0.00020 Ang.
PLAT143_ALERT_4_G s.u. on c - Axis Small or Missing .............    0.00010 Ang.
PLAT171_ALERT_4_G The CIF-Embedded .res File Contains EADP Records          1 Report
PLAT172_ALERT_4_G The CIF-Embedded .res File Contains DFIX Records         18 Report
PLAT177_ALERT_4_G The CIF-Embedded .res File Contains DELU Records          2 Report
PLAT178_ALERT_4_G The CIF-Embedded .res File Contains SIMU Records          6 Report
PLAT186_ALERT_4_G The CIF-Embedded .res File Contains ISOR Records          3 Report
PLAT188_ALERT_3_G A Non-default SIMU Restraint Value has been used     0.0100 Report
PLAT188_ALERT_3_G A Non-default SIMU Restraint Value has been used     0.0100 Report
PLAT188_ALERT_3_G A Non-default SIMU Restraint Value has been used     0.0200 Report
PLAT188_ALERT_3_G A Non-default SIMU Restraint Value has been used     0.0200 Report
PLAT192_ALERT_3_G A Non-default DELU Restraint Value for SecondPar     0.0200 Report
PLAT242_ALERT_2_G Low    'MainMol' Ueq as Compared to Neighbors of       C00Q Check
PLAT242_ALERT_2_G Low    'MainMol' Ueq as Compared to Neighbors of       C010 Check
PLAT242_ALERT_2_G Low    'MainMol' Ueq as Compared to Neighbors of       C011 Check
PLAT242_ALERT_2_G Low    'MainMol' Ueq as Compared to Neighbors of       C019 Check
PLAT242_ALERT_2_G Low    'MainMol' Ueq as Compared to Neighbors of       C01D Check
PLAT242_ALERT_2_G Low    'MainMol' Ueq as Compared to Neighbors of       C01E Check
PLAT299_ALERT_4_G Atom Site Occupancy Constrained at ............         0.5 Check
              H01C    H01D    H01E    H01F    H01G    H01H
PLAT300_ALERT_4_G Atom Site Occupancy of C00G       Constrained at       0.75 Check
PLAT300_ALERT_4_G Atom Site Occupancy of C1         Constrained at       0.25 Check
PLAT301_ALERT_3_G Main Residue  Disorder .............(Resd    1)          2% Note
PLAT412_ALERT_2_G Short Intra XH3 .. XHn     H01A     ..H01F     .       1.91 Ang.
                                                  x,y,z  =      1_555 Check
PLAT720_ALERT_4_G Number of Unusual/Non-Standard Labels ..........         62 Note
              Ag01    Ag02    Ag03    S004    F005    F006    F007    F008
              F009    F00A    F00B    F00C    F00D    F00E    F00F    C00G
              F00H    F00I    N00J    F00K    F00L    F00M    N00N    N00O
              N00P    C00Q    N00R    F00S    C00T    H00T    O00U    N00V
              C00W    C00X    C00Y    C00Z    H00Z    C010    C011    C012
              F013    C014    H014    C015    C016    C017    C018    H018
              C019    C01A    H01A    C01B    H01B    C01C    H01C    H01D
              H01E    H01F    H01G    H01H    C01D    C01E
PLAT860_ALERT_3_G Number of Least-Squares Restraints ............         140 Note
PLAT883_ALERT_1_G Absent Datum for _atom_sites_solution_primary ..     Please Do !
PLAT912_ALERT_4_G Missing # of FCF Reflections Above STh/L=  0.600         93 Note
PLAT915_ALERT_3_G No Flack x Check Done: Low Friedel Pair Coverage         45 %
PLAT933_ALERT_2_G Number of HKL-OMIT Records in Embedded .res File          1 Note
               12 12 -2,
PLAT941_ALERT_3_G Average HKL Measurement Multiplicity ..........         4.3 Low
PLAT969_ALERT_5_G The 'Henn et al.' R-Factor-gap value ..........       4.128 Note
              Predicted wR2: Based on SigI**2  4.69 or SHELX Weight 16.84
PLAT978_ALERT_2_G Number C-C Bonds with Positive Residual Density.          1 Info
```

---

0 **ALERT level A** = Most likely a serious problem - resolve or explain
2 **ALERT level B** = A potentially serious problem, consider carefully

```
 13 ALERT level C = Check. Ensure it is not caused by an omission or oversight
 35 ALERT level G = General information/check it is not something unexpected

  2 ALERT type 1 CIF construction/syntax error, inconsistent or missing data
 21 ALERT type 2 Indicator that the structure model may be wrong or deficient
 13 ALERT type 3 Indicator that the structure quality may be low
 13 ALERT type 4 Improvement, methodology, query or suggestion
  1 ALERT type 5 Informative message, check
```

Validation response form

Please find below a validation response form (VRF) that can be filled in and pasted into your CIF.

```
# start Validation Reply Form
_vrf_PLAT042_lcz086
;
PROBLEM: Calc. and Reported MoietyFormula Strings  Differ     Please Check
RESPONSE: ...
;
_vrf_PLAT090_lcz086
;
PROBLEM: Poor Data / Parameter Ratio (Zmax > 18) ........       7.24 Note
RESPONSE: ...
;
_vrf_PLAT213_lcz086
;
PROBLEM: Atom F00L            has ADP max/min Ratio .....        3.5 prolat
RESPONSE: ...
;
_vrf_PLAT234_lcz086
;
PROBLEM: Large Hirshfeld Difference F008     --C019     .       0.22 Ang.
RESPONSE: ...
;
_vrf_PLAT241_lcz086
;
PROBLEM: High   'MainMol' Ueq as Compared to Neighbors of       O00U Check
RESPONSE: ...
;
_vrf_PLAT906_lcz086
;
PROBLEM: Large K Value in the Analysis of Variance ......      4.095 Check
RESPONSE: ...
;
_vrf_PLAT911_lcz086
;
PROBLEM: Missing FCF Refl Between Thmin & STh/L=    0.600          3 Report
RESPONSE: ...
;
_vrf_PLAT972_lcz086
;
PROBLEM: Check Calcd Resid. Dens.  0.38Ang From O00U          -1.65 eA-3
RESPONSE: ...
;
_vrf_PLAT977_lcz086
```

```
;
PROBLEM: Check Negative Difference Density on H014      .      -0.31 eA-3
RESPONSE: ...
;
# end Validation Reply Form
```

It is advisable to attempt to resolve as many as possible of the alerts in all categories. Often the minor alerts point to easily fixed oversights, errors and omissions in your CIF or refinement strategy, so attention to these fine details can be worthwhile. It is up to the individual to critically assess their own results and, if necessary, seek expert advice.

PLATON version of 17/09/2025; check.def file version of 17/09/2025

---

duplicate check

No duplication found

Datablock lcz086 - ellipsoid plot

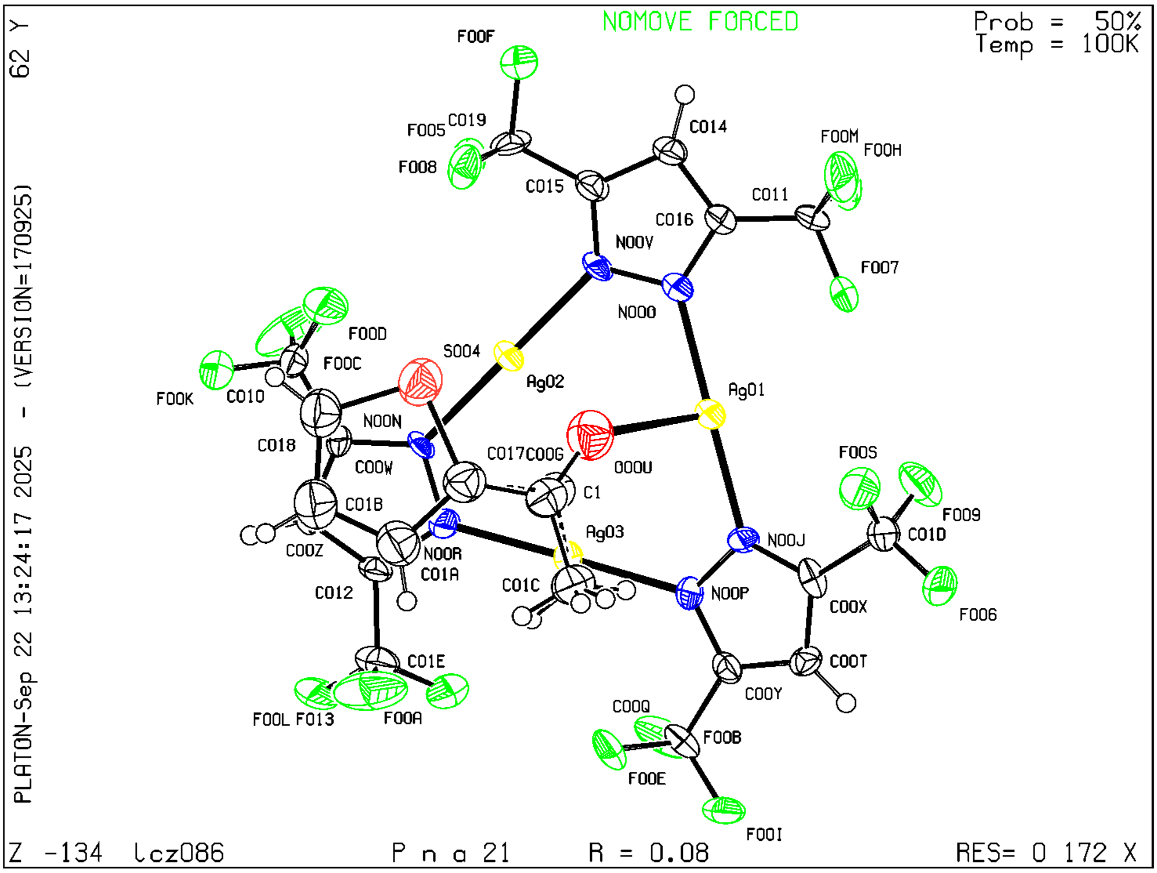

Supplement: Supplementary file 2 — Supporting File 2: anie72462‐sup‐0002‐DataFile.zip. [file ANIE-65-e1218503-s002.zip › supplementary_materials_crystallographic_data submit.zip!/cif_and_checkcif(1-114)/Ag3Pz3_86/Ag3Pz3_86_checkcif.pdf]
